# Characterization of the complete mitochondrial genome of *Pennisetum giganteum* A. Rich. (Poaceae)

**DOI:** 10.1080/23802359.2025.2550607

**Published:** 2025-08-23

**Authors:** Man Zhang, Xiaojun Wu, Kaiqiang Fang, Haobo Wang, Junchao You, Xiangdong Chen

**Affiliations:** ^a^ Wheat Research Center, Henan Institute of Science and Technology, Xinxiang, China; ^b^ State Key Laboratory of High-Efficiency Production of Wheat-Maize Double Cropping, Xinxiang, China; ^c^ Henan Key Laboratory of Hybrid Wheat, Xinxiang, China

**Keywords:** Poaceae, C_4_ plant, mitogenomics, mitochondrial genome assembly, phylogenomics, Panicoideae

## Abstract

The complete mitochondrial genome of *Pennisetum giganteum*, an African species, was sequenced and characterized for the first time. The genome is 491,311 bp long and contains 53 unique genes. Phylogenetic analysis based on these genes using maximum likelihood revealed that *P. giganteum* is most closely related to Saccharum officinarum and most distantly related to *Oryza sativa*. This study provides essential genomic data that enhances the understanding of the taxonomy and evolutionary relationships of this versatile and high-yielding species.

## Introduction

*Pennisetum giganteum* A. Rich. (Poaceae) is a perennial C_4_ grass native to Africa, widely distributed in tropical to subtropical regions due to its adaptability to warm climates (Fulkerson et al. ^2007^; Xing et al. ^2023^). As a member of the genus *Pennisetum*, *P. giganteum* has significant ecological and agricultural value, noted for rapid growth, high biomass yield, and applications in sustainable agriculture (Samson et al. ^2005^; Hayat et al. ^2020^). However, genomic resources for *P. giganteum* remain scarce, particularly for organellar genomes. While chloroplast data exist for close relatives like *Pennisetum glaucum* (Xu et al. ^2019^), no mitochondrial genome has been reported for any *Pennisetum* species (Zheng et al. ^2023^).

Mitochondrial genomes are pivotal for resolving plant phylogenies due to structural complexity and variable evolutionary rates (Zardoya ^2020^). In *Pennisetum*, taxonomic relationships are complicated by hybridization and polyploidization (Dos Reis et al. ^2014^; Xing et al. ^2023^). Although nuclear and chloroplast markers have been used (Xu et al. ^2019^; Zheng et al. ^2023^), mitochondrial data are critical for evolutionary insights (Shearman et al. ^2016^; Wu et al. ^2022^). Here, we present the first complete mitochondrial genome of *P. giganteum*, assembled using Illumina and Nanopore sequencing. This work addresses a genomic gap and provides foundational data for studies on mitochondrial evolution, C_4_ photosynthesis (Fan et al. ^2022^), and phylogenetics in this key genus.

## Materials and methods

### Plant materials

**Figure 1.**
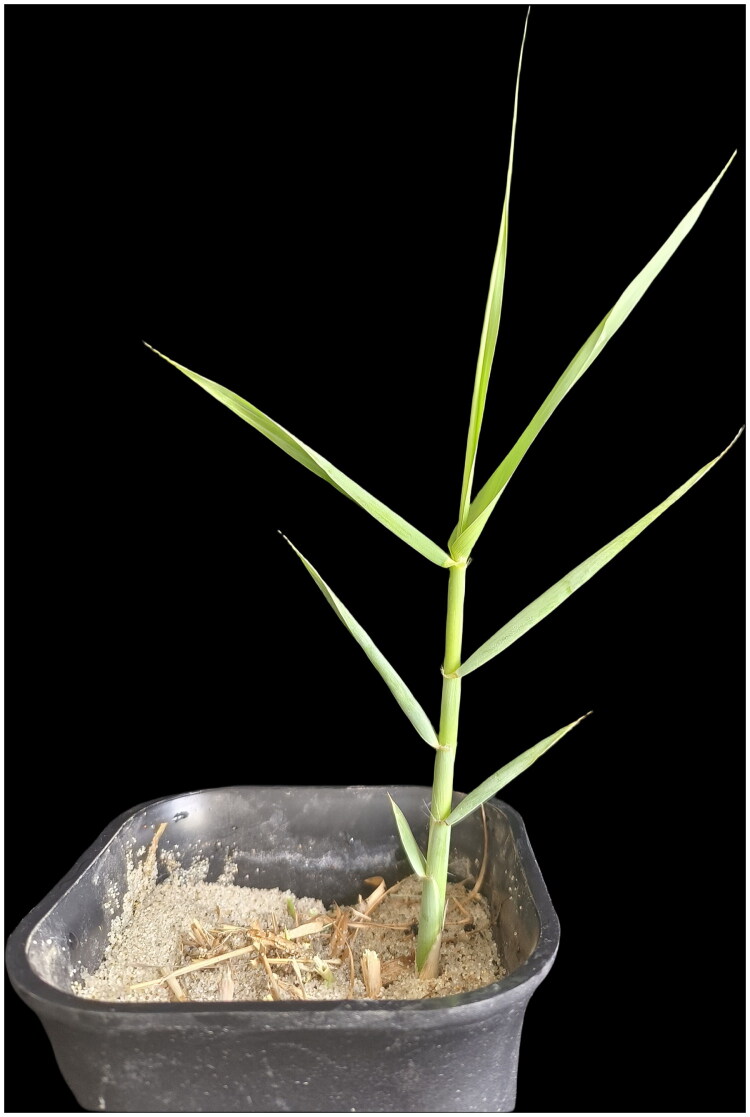
Morphology features of *Pennisetum giganteum*. The seedling stage of *Pennisetum giganteum* is characterized by erect plants and alternate leaves. The photos of *Pennisetum giganteum* were taken by Man Zhang at the Botanical Garden of the Center of Wheat Research of Henan Institute of Science and Technology, Xinxiang, Henan, China.

The *P. giganteum* was used in this study. The voucher specimen was identified by researchers and deposited at the Herbarium of Henan Institute of Science and Technology, Xinxiang, China, under voucher number XM-W145 (contact: Zhengang Ru; rzgh5819@163.com). Fresh roots of *P. giganteum* were collected from the Botanical Garden of the Wheat Research Center, Xinxiang City, Henan Province, China (113°52′E, 35°30′N) by Man Zhang (Figure 1).

### DNA sequencing, genome assembly, and annotation

A genomic DNA sample was isolated from the roots with a Plant Tissue Mitochondrial DNA Extraction Kit (Genmed Scientific Inc., Arlington, MA, USA) according to the manufacturer’s instructions, and quality control was subsequently carried out on the purified DNA samples. The quality of DNA was assessed using Qubit3.0 and 1% agarose gel electrophoresis. Highly qualified DNA sample (OD260/280 = 1.8 ∼ 2.0, >6ug) was utilized to construct the fragment library.

To obtain full-length mitochondrial genome sequences, we used both short-read (Illumina NovaSeq 6000 platform) and long-read sequencing (Nanopore sequencing platform) technologies in this study. The raw paired-end reads were trimmed and quality controlled by Trimmomatic with parameters (SLIDINGWINDOW:4:15 MINLEN:75) (Bolger et al. ^2014^). Clean data obtained through the above quality control processes was used for further analysis.

The Illumina NovaSeq 6000 short reads (6.3 Gb) were used to evaluate the complexity of the genome and correct the Nanopore long reads. First, we used ABySS 2.2.0 (Simpson et al. ^2009^) to perform genome assembly with multiple-kmer parameters and received the optimal results of the assembly. Secondly, blasR (Chaisson and Tesler ^2012^) was used to map the preliminary assembly results to the Nanopore long reads (6.3 Gb). Then, SPAdes-3.15.5 (Prjibelski et al. ^2020^) was used to assemble them (Nanopore and NGS data) together to construct contigs (Scaffolds). Finally, GapCloser v1.12 (Xu et al. ^2020^) software was applied to fill the remaining local inner gaps and correct the single-base polymorphism for the final assembly results.

The mitochondrial genes were annotated through homology alignments and de novo prediction. Gene prediction for tRNAs was performed using tRNAscan-SE 2.0.4 (Chan and Lowe ^2019^) with default parameters. For rRNA and protein-coding genes (PCGs), the assembled mitochondrial genome of *P. giganteum* (query) was aligned against the complete mitochondrial genome of *Saccharum officinarum* (reference database; GenBank: LC107874) *via* BLASTn (BLAST + 2.9.0; Camacho et al. ^2009^). Homologous regions meeting thresholds of ≥80% sequence identity and ≥90% coverage were retained, and gene boundaries were further refined using ORFfinder (v0.4.1; Sayers et al. ^2024^).

Finally, the circular mitochondrial genome assembly and split gene identification were performed and validated using the Plant Mitochondrial Genomes Map (PMGmap) v2.0 (Zhang et al. ^2024^), a tool specialized in structural annotation based on sequence conservation and break-join modeling.

The annotated mitogenome was submitted to the GenBank database under accession number PQ768538. Then all gene models were analyzed by BLASTp (Camacho et al. ^2009^) against the non-redundant (NR) protein database (Sayers et al. ^2024^), SwissProt (The UniProt Consortium ^2019^), KEGG (Kanehisa and Goto ^2000^), Gene Ontology (GO) (The Gene Ontology Consortium ^2000^), and Clusters of Orthologous Groups (COG) (Tatusov et al. ^2000^) for functional annotation.

To validate the assembly completeness and uniformity, sequencing reads were mapped back to the mitochondrial genome using BWA-MEM v0.7.17 (Li ^2013^) with default parameters. The coverage depth across the genome was calculated using SAMtools v1.18 (Danecek et al. ^2021^) and visualized as a coverage plot (Figure S1) *via* the Integrative Genomics Viewer (IGV) v2.19.1 (Thorvaldsdóttir et al. ^2013^). The average coverage depth of 284.04× indicates sufficient sequencing depth to ensure high-confidence assembly and highlights uniform read distribution, which minimizes potential gaps or misassemblies (Figure S1). Coverage analysis also helps identify repetitive regions or structural variations that may challenge assembly algorithms.

### Phylogenetic tree construction

To determine the molecular position of *P. giganteum* with other closely related species, phylogenetic analyses were performed using 11 complete mitochondrial genome sequences (comprising 34 PCGs, 16 tRNAs, and three rRNAs). First, global alignment was conducted with MUMmer v3.23 (Delcher et al. ^2002^) to identify syntenic blocks. Local alignment refinement was performed using BLAT v36 (Kent ^2002^) with default parameters. To ensure alignment quality, ambiguous and divergent regions were filtered using Gblocks v0.91b (Castresana ^2000^) under strict parameters (-t = d -b4 = 5 -b5 = h), retaining only highly conserved blocks suitable for phylogenetic inference. The best-fit nucleotide substitution model (GTR+I + G) was selected using jModelTest 2.1.10 (Darriba et al. ^2012^), and maximum-likelihood trees were constructed with PhyML 3.0 (Guindon et al. ^2010^) using 1,000 bootstrap replicates. *Oryza sativa* Japonica Group (accession BA000029.3) was selected as the outgroup, as its placement in the evolutionarily distinct Oryzoideae subfamily provides a robust phylogenetic contrast to the Panicoideae subfamily (containing *Pennisetum*), and its well-annotated mitochondrial genome is widely adopted in Poaceae studies.

The assembled mitochondrial genome (491,311 bp; GenBank ID: PQ768538) contains 34 protein-coding genes, 16 tRNA genes, and 3 rRNA genes. This circular map displays the following features from the innermost to the outermost ring: Relationships between dispersed repeats; Distribution of dispersed repeats along the genome (direct repeats shown in yellow, inverted repeats in green).

Distribution of tandem repeats; GC content across the genome; Scale axis; Genes encoded on the negative strand, genes encoded on the positive strand.

## Results

The *P. giganteum* mitochondrial genome (GenBank ID: PQ768538) was assembled into a single, circular sequence with a length of 491,311 bp and a GC content of 43.81%.

**Figure 2.**
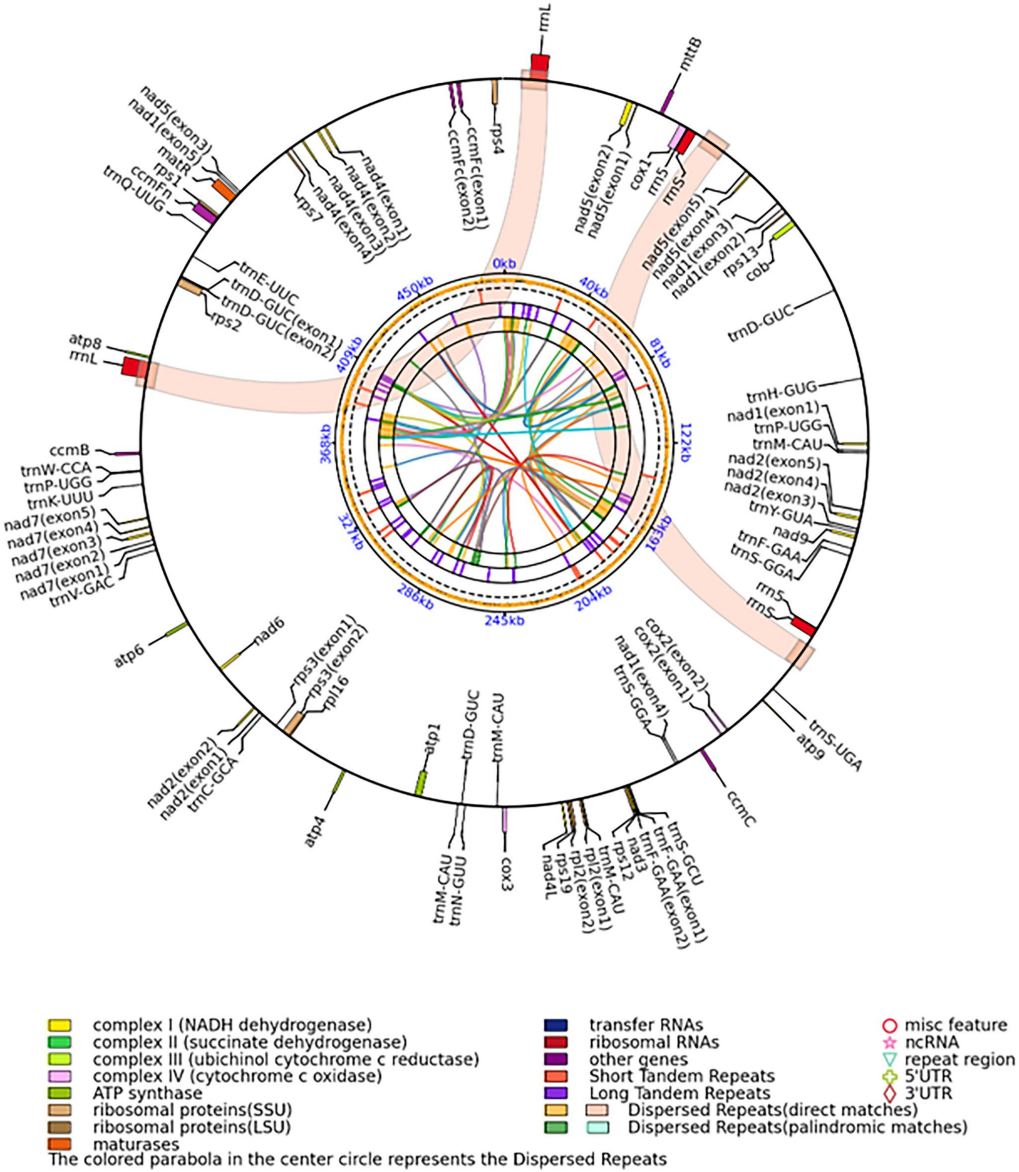
Mitochondrial genome map of *Pennisetum giganteum* constructed with PMGmap.

The mitochondrial genome of *P. giganteum* includes 34 PCGs, 16 tRNA genes, and three rRNA genes (Figure 2, Table S1). Most of the PCGs have an ATG start codon, except for nad1 and nad4L, which have an ACG start codon. There are two types of termination codons in the PCGs: TAA and TAG, which correspond to 15 and 19 genes, respectively. Among the PCGs, eight were *cis*-spliced genes, six of which contained one intron (*ccmFc*, *cox2*, *rpl2*, *rps3*, *trnD-GUC*, *trnF-GAA*), one contained three introns (*nad4*), and one contained four introns (*nad7*). *nad1*, *nad2*, and *nad5* were *trans*-spliced genes, all of which contained four introns (Figure S2).

The 16 tRNAs ranged from 68 to 89 bp in length. *rrn5*, *rrnL*, and *rrnS* genes were 120 bp, 3535 bp, and 1967 bp in length, respectively. There were 17 gene duplications, including 9 PCGs, five tRNA genes, and three rRNA genes.

**Figure 3.**
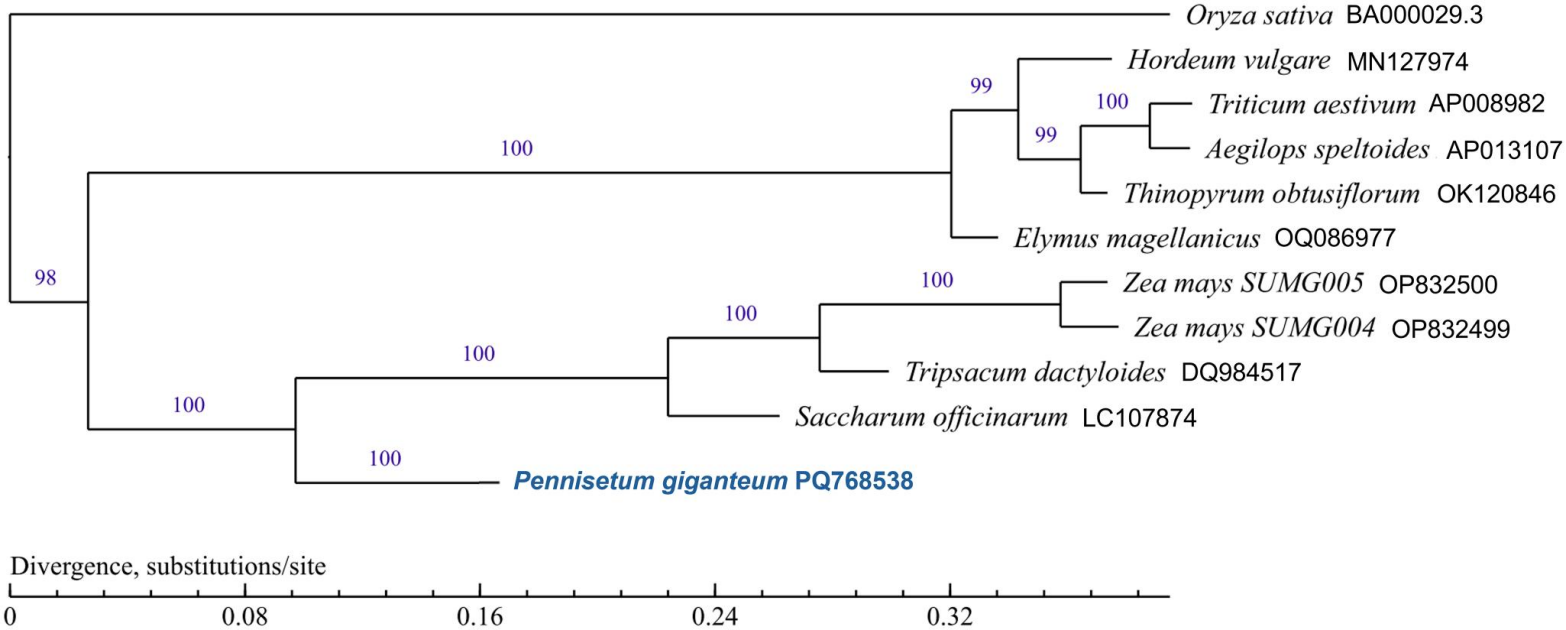
The maximum likelihood (ML) phylogenetic tree was constructed using the complete mitochondrial genome of 11 species. The following sequences were used: *Pennisetum giganteum* (GenBank: PQ768538), *Oryza sativa* Japonica Group (GenBank: BA000029.3; Notsu et al. ^2002^), *Hordeum vulgare* (GenBank: MN127974), *Triticum aestivum* (GenBank: AP008982; Ogihara et al. ^2005^), *Aegilops speltoides* (GenBank: AP013107), *Thinopyrum obtusiflorum* (GenBank: OK120846; Wu et al. ^2022^), *Elymus magellanicus* (GenBank: OQ086977; Chen et al. ^2023^), *Zea mays* voucher SUMG 004 (GenBank: OP832499), *Zea mays* voucher SUMG 005 (GenBank: OP832500), *Tripsacum dactyloides* (GenBank: DQ984517), *Saccharum officinarum* (GenBank: LC107874; Shearman et al. ^2016^). The phylogenetic tree was rooted with the outgroup (*Oryza* sativa), with branch lengths scaled to substitutions per site. Numbers next to each node indicate bootstrap support values.

This study analyzed the phylogenetic relationships of 11 species using the complete mitochondrial genome sequence (Figure 3). As designated a priori for phylogenetic contrast, Oryza sativa Japonica Group (BA000029.3) formed a distinct basal branch serving as the outgroup. *P. giganteum* is most closely related to *S. officinarum* (LC107874), followed by *Tripsacum dactyloides* (DQ984517), *Zea mays* voucher SUMG 004 (OP832499), and *Zea mays* voucher SUMG 005 (OP832500). The above five species are grouped into one clade. Another large clade also contains five species: *Elymus magellanicus* (OQ086977), *Thinopyrum obtusiflorum* (OK120846), *Aegilops speltoides* (AP013107), *Triticum aestivum* (AP008982), and *Hordeum vulgare* (MN127974). Results from distant species are presented as supplementary references, with core conclusions drawn from intra-Panicoideae relationships.

## Discussion and conclusion

Compared to animal mitochondria, plant mitochondrial genomes exhibit greater structural complexity and variability. Consequently, research has predominantly focused on chloroplasts, leaving many plant mitochondrial genomes unresolved (Zardoya ^2020^). This study presents the first complete assembly and annotation of the mitochondrial genome of *P. giganteum*, marking the first reported mitochondrial genome for any species within the genus *Pennisetum*.

The assembly and annotation of the *P. giganteum* mitochondrial genome (491,311 bp) provide critical insights into its genomic architecture. Its moderate GC content (43.81%) aligns closely with mitochondrial genomes of other Poaceae species, such as *S. officinarum* (43.9%) and *Zea mays* (43.9%), suggesting conserved compositional stability among C_4_ grasses (Clifton et al. ^2004^; Xing et al. ^2023^; Li et al. ^2025^). Notably, the presence of atypical start codons (e.g. ACG for *nad1*) mirrors findings in *Elymus magellanicus* (Chen et al. ^2023^) and *Saccharum* species (Zhou et al. ^2022^), highlighting potential evolutionary constraints in mitochondrial gene regulation.

Mitochondrial PCGs exhibit significantly slower evolutionary rates, particularly in synonymous substitutions (Wu et al. ^2020^). This high conservation makes them suitable for resolving deep-level phylogenetic relationships, contrasting with chloroplast genomes better suited for lower taxonomic ranks (Lan et al. ^2024^).

The coexistence of *cis*-spliced and *trans*-spliced genes underscores intricate RNA processing in *P. giganteum*, aligning with mitochondrial genome plasticity observed in other polyploid grasses (Guo et al. ^2020^). This characterization provides foundational insights into its potential role in supporting C_4_ photosynthetic evolution.

Phylogenetic analysis revealed *P. giganteum*’s closest relationship with *S. officinarum* (a C_4_ grass), consistent with morphological and ecological similarities, while showing marked divergence from C_3_ species like *O. sativa*. This mitochondrial genome serves as a crucial reference for resolving taxonomic uncertainties, studying polyploid mitochondrial evolution, and developing molecular markers. These findings establish a foundation for future investigations into C_4_ plant mitochondrial-chloroplast interactions and stress tolerance genetics. Integrating mitogenomic data with nuclear and chloroplast genomes will enhance understanding of adaptive evolution in this genus.

## Supplementary Material

Supplementary materials.docx

## Data Availability

The genome sequence data that support the findings of this study are openly available in GenBank of NCBI at (https://www.ncbi.nlm.nih.gov/), reference number PQ768538. The associated BioProject, Bio-Sample, and SRA numbers are PRJNA1196327, SAMN45421721, SRR31666584 (Nanopore) and SRR31666585 (NovaSeq), respectively.

## References

[CIT0001] Bolger AM, Lohse M, Usadel B. 2014. Trimmomatic: a flexible trimmer for Illumina sequence data. Bioinformatics. 30(15):2114–2120. doi:10.1093/bioinformatics/btu170. 24695404 PMC4103590

[CIT0002] Camacho C, Coulouris G, Avagyan V, Ma N, Papadopoulos J, Bealer K, Madden TL. 2009. BLAST+: architecture and applications. BMC Bioinform. 10(1):421. doi:10.1186/1471-2105-10-421. PMC2803857 20003500

[CIT0003] Castresana J. 2000. Selection of conserved blocks from multiple alignments for their use in phylogenetic analysis. Mol Biol Evol. 17(4):540–552. doi:10.1093/oxfordjournals.molbev.a026334. 10742046

[CIT0004] Chaisson MJ, Tesler G. 2012. Mapping single molecule sequencing reads using basic local alignment with successive refinement (BLASR): application and theory. BMC Bioinformatics. 13(1):238. doi:10.1186/1471-2105-13-238. 22988817 PMC3572422

[CIT0005] Chan PP, Lowe TM. 2019. tRNAscan-SE: searching for tRNA genes in genomic sequences. Methods Mol Biol. 1962:1–14. doi:10.1007/978-1-4939-9173-0_1. 31020551 PMC6768409

[CIT0006] Chen XD, Wu XJ, Zhang JL, Zhang M, You JC, Ru ZG. 2023. Characterization of the complete mitochondrial genome of *Elymus magellanicus* (É. Desv.) Á. Löve (Poaceae, Pooideae). Mitochondrial DNA B Resour. 8(8):795–798. doi:10.1080/23802359.2023.2238931. 37545550 PMC10399486

[CIT0007] Clifton SW, Minx P, Fauron CM, Gibson M, Allen JO, Sun H, Thompson M, Barbazuk WB, Kanuganti S, Tayloe C, et al. 2004. Sequence and comparative analysis of the maize NB mitochondrial genome. Plant Physiol. 136(3):3486–3503. doi:10.1104/pp.104.044602. 15542500 PMC527149

[CIT0008] Danecek P, Bonfield JK, Liddle J, Marshall J, Ohan V, Pollard MO, Whitwham A, Keane T, McCarthy SA, Davies RM, et al. 2021. Twelve years of SAMtools and BCFtools. Gigascience. 10(2):giab008. doi:10.1093/gigascience/giab008. 33590861 PMC7931819

[CIT0009] Darriba D, Taboada GL, Doallo R, Posada D. 2012. jModelTest 2: more models, new heuristics and parallel computing. Nat Methods. 9(8):772–772. doi:10.1038/nmeth.2109. PMC4594756 22847109

[CIT0010] Delcher AL, Phillippy A, Carlton J, Salzberg SL. 2002. Fast algorithms for large-scale genome alignment and comparison. Nucleic Acids Res. 30(11):2478–2483. doi:10.1093/nar/30.11.2478. 12034836 PMC117189

[CIT0011] Dos Reis GB, Mesquita AT, Torres GA, Andrade-Vieira LF, Pereira AV, Davide LC. 2014. Genomic homeology between *Pennisetum purpureum* and *Pennisetum glaucum* (Poaceae). Comp Cytogenet. 8(3):199–209. doi:10.3897/CompCytogen.v8i3.7732. 25349671 PMC4205489

[CIT0012] Fan YZ, Asao S, Furbank RT, Caemmerer SV, Day DA, Tcherkez G, Sage TL, Sage RF, Atkin OK. 2022. The crucial roles of mitochondria in supporting C4 photosynthesis. New Phytol. 233(3):1083–1096. doi:10.1111/nph.17818. 34669188

[CIT0013] Fulkerson WJ, Neal JS, Clark CF, Horadagoda A, Nandra KS, Barchia I. 2007. Nutritive value of forage species grown in the warm temperate climate of Australia for dairy cows: grasses and legumes. Livest Sci. 107(2-3):253–264. doi:10.1016/j.livsci.2007.04.013.

[CIT0014] Gene Ontology Consortium. 2000. Gene ontology: tool for the unification of biology. Nat Genet. 25(1):25–29. doi:10.1038/75556. 10802651 PMC3037419

[CIT0015] Guindon S, Dufayard J-F, Lefort V, Anisimova M, Hordijk W, Gascuel O. 2010. New algorithms and methods to estimate maximum-likelihood phylogenies: assessing the performance of PhyML 3.0. Syst Biol. 59(3):307–321. doi:10.1093/sysbio/syq010. 20525638

[CIT0016] Guo W, Zhu A, Fan W, Adams RP, Mower JP. 2020. Extensive shifts from *cis*- to *trans*-splicing of gymnosperm mitochondrial introns. Mol Biol Evol. 37(6):1615–1620. doi:10.1093/molbev/msaa029. 32027368

[CIT0017] Hayat K, Zhou YF, Menhas S, Bundschuh J, Hayat S, Ullah A, Wang J, Chen XF, Zhang D, Zhou P. 2020. *Pennisetum giganteum*: an emerging salt accumulating/tolerant non-conventional crop for sustainable saline agriculture and simultaneous phytoremediation. Environ Pollut. 265(Pt A):114876. doi:10.1016/j.envpol.2020.114876. 32512425

[CIT0018] Kanehisa M, Goto S. 2000. KEGG: kyoto encyclopedia of genes and genomes. Nucleic Acids Res. 28(1):27–30. doi:10.1093/nar/28.1.27. 10592173 PMC102409

[CIT0019] Kent WJ. 2002. BLAT—the BLAST-like alignment tool. Genome Res. 12(4):656–664. doi:10.1101/gr.229202. 11932250 PMC187518

[CIT0020] Lan WX, Mo Q, Jin MM, Wen YH, Yang MQ, Ma H, Huang HQ, Huang MJ. 2024. Exploring the phylogenetic framework and trait evolution of Impatiens through chloroplast genome analysis. BMC Plant Biol. 24(1):1218. doi:10.1186/s12870-024-05964-y. 39702025 PMC11660898

[CIT0021] Li H. 2013. Aligning sequence reads, clone sequences and assembly contigs with BWA-MEM. *arXiv:1303.3997*. doi:10.48550/arXiv.1303.3997.

[CIT0022] Li Y, Li S, Hua X, Wang M, Zhang Y, Chen R, Liu L, Zhang J, Mei Y, Wu J, et al. 2025. Mitochondrial genome structural variants and candidate cytoplasmic male sterility-related gene in sugarcane. BMC Genomics. 26(1):16. doi:10.1186/s12864-025-1234-9. 39794692 PMC11724576

[CIT0023] Notsu Y, Masood S, Nishikawa T, Kubo N, Akiduki G, Nakazono M, Hirai A, Kadowaki K. 2002. The complete sequence of the rice (*Oryza sativa* L.) mitochondrial genome: frequent DNA sequence acquisition and loss during the evolution of flowering plants. Mol Genet Genomics. 268(4):434–445. doi:10.1007/s00438-002-0767-1. 12471441

[CIT0024] Ogihara Y, Yamazaki Y, Murai K, Kanno A, Terachi T, Shiina T, Miyashita N, Nasuda S, Nakamura C, Mori N, et al. 2005. Structural dynamics of cereal mitochondrial genomes as revealed by complete nucleotide sequencing of the wheat mitochondrial genome. Nucleic Acids Res. 33(19):6235–6250. doi:10.1093/nar/gki925. 16260473 PMC1275586

[CIT0025] Prjibelski AD, Vasilinetc I, Bankevich A, Gurevich A, Krivosheeva T, Nurk S, Antipov D. 2020. Using SPAdes de novo assembler. Curr Protoc Bioinformatics. 70(1):e102. doi:10.1002/cpbi.102. 32559359

[CIT0026] Samson R, Mani S, Boddey R, Sokhansanj S, Quesada D, Urquiaga S, Reis V, Lem CH. 2005. The potential of C_4_ perennial grasses for developing a global BIOHEAT industry. Crit Rev Plant Sci. 24(5-6):461–495. doi:10.1080/07352680500316508.

[CIT0027] Sayers EW, Beck J, Bolton EE, Brister JR, Chan J, Comeau DC, Connor R, DiCuccio M, Farrell CM, Feldgarden M, et al. 2024. Database resources of the National Center for Biotechnology Information. Nucleic Acids Res. 52(D1):D33–D43. doi:10.1093/nar/gkad1044. 37994677 PMC10767890

[CIT0028] Shearman JR, Sonthirod C, Naktang C, Pootakham W, Yoocha T, Sangsrakru D, Jomchai N, Tragoonrung S, Tangphatsornruang S. 2016. The two chromosomes of the mitochondrial genome of a sugarcane cultivar: assembly and recombination analysis using long PacBio reads. Sci Rep. 6(1):31533. doi:10.1038/srep31533. 27530092 PMC4987617

[CIT0029] Simpson JT, Wong K, Jackman SD, Schein JE, Jones SJ, Birol I. 2009. ABySS: a parallel assembler for short read sequence data. Genome Res. 19(6):1117–1123. doi:10.1101/gr.089532.108. 19251739 PMC2694472

[CIT0030] Tatusov RL, Galperin MY, Natale DA, Koonin EV. 2000. The COG database: a tool for genome-scale analysis of protein functions and evolution. Nucleic Acids Res. 28(1):33–36. doi:10.1093/nar/28.1.33. 10592175 PMC102395

[CIT0031] Thorvaldsdóttir H, Robinson JT, Mesirov JP. 2013. Integrative Genomics Viewer (IGV): high-performance genomics data visualization and exploration. Brief Bioinform. 14(2):178–192. doi:10.1093/bib/bbs017. 22517427 PMC3603213

[CIT0032] UniProt Consortium. 2019. UniProt: a worldwide hub of protein knowledge. Nucleic Acids Res. 47(D1):D506–D515. doi:10.1093/nar/gky1049. 30395287 PMC6323992

[CIT0033] Wu XJ, Hu XG, Chen XD, Zhang JL, Ren CC, Song LT, Fang F, Dong N, Hu TZ, Ru ZG. 2022. Sequencing and characterization of the complete mitochondrial genome of *Thinopyrum obtusiflorum* (DC.) Banfi, 2018 (Poaceae). Mitochondrial DNA B Resour. 7(3):539–540. doi:10.1080/23802359.2022.2054378. 35356795 PMC8959512

[CIT0034] Wu Z, Waneka G, Sloan DB. 2020. The tempo and mode of angiosperm mitochondrial genome divergence inferred from intraspecific variation in Arabidopsis thaliana. G3 (Bethesda). 10(3):1077–1086. doi:10.1534/g3.119.401023. 31964685 PMC7056966

[CIT0035] Xing LS, Wang MJ, He Q, Zhang HY, Liang HF, Zhou QH, Liu Y, Liu Z, Wang Y, Du CL, et al. 2023. Differential subgenome expression underlies biomass accumulation in allotetraploid *Pennisetum giganteum*. BMC Biol. 21(1):161. doi:10.1186/s12915-023-01643-w. 37480118 PMC10362693

[CIT0036] Xu J, Song Y, Jing XY, Li MF. 2019. Characterization of the complete chloroplast genome sequence of *Pennisetum glaucum* and its phylogenetic implications. Mitochondrial DNA B Resour. 4(2):3764–3765. doi:10.1080/23802359.2019.1668312. 33366180 PMC7707298

[CIT0037] Xu MY, Guo LD, Gu SQ, Wang O, Zhang R, Peters BA, Fan GY, Liu X, Xu X, Deng L, et al. 2020. TGS-GapCloser: a fast and accurate gap closer for large genomes with low coverage of error-prone long reads. Gigascience. 9(9):giaa094. doi:10.1093/gigascience/giaa094. 32893860 PMC7476103

[CIT0038] Zardoya R. 2020. Recent advances in understanding mitochondrial genome diversity. F1000Res. 9:270. doi:10.12688/f1000research.21490.1. PMC7194472 32399193

[CIT0039] Zhang X, Chen H, Ni Y, Wu B, Li J, Burzyński A, Liu C.,. 2024. Plant mitochondrial genome map (PMGmap): a software tool for the comprehensive visualization of coding, noncoding and genome features of plant mitochondrial genomes. Mol Ecol Resour. 24(5):e13952. doi:10.1111/1755-0998.13952. 38523350

[CIT0040] Zheng HK, Wang BY, Hua XT, Gao RT, Wang YH, Zhang ZX, Zhang YX, Mei J, Huang YJ, Huang YM, et al. 2023. A near-complete genome assembly of the allotetrapolyploid *Cenchrus fungigraminus* (JUJUNCAO) provides insights into its evolution and C_4_ photosynthesis. Plant Commun. 4(5):100633. doi:10.1016/j.xplc.2023.100633. 37271992 PMC10504591

[CIT0041] Zhou D, Liu Y, Yao J, Yin Z, Wang X, Xu L, Que Y, Mo P, Liu X. 2022. Characterization and phylogenetic analyses of the complete mitochondrial genome of sugarcane (*Saccharum* spp. Hybrids) line A1. Diversity. 14(5):333. doi:10.3390/d14050333.

