# Supplementary material for: Characterization of the complete mitochondrial genome of Pennisetum giganteum A. Rich. (Poaceae)
Source: Mitochondrial DNA B Resour. 2025 Aug 23;10(9):868–73. doi: 10.1080/23802359.2025.2550607 (PMC12377103; doi:10.1080/23802359.2025.2550607)
Supplement: Supplementary materials.docx [file TMDN_A_2550607_SM6937.docx]

**Supplementary material**

**[Characterization of the complete](https://www.tandfonline.com/doi/full/10.1080/23802359.2021.1981788)** **[mitochondrial genome of](https://www.tandfonline.com/doi/full/10.1080/23802359.2021.1981788)** ***Pennisetum giganteum* A. Rich*.* (Poaceae)**

Man Zhang^a,b,c^, Xiaojun Wu^a,b,c^, Kaiqiang Fang^a,b,c^, Haobo Wang^a,b,c^, Junchao You^a,b,c^, and Xiangdong Chen^a,b,c^

^a^ Wheat Research Center, Henan Institute of Science and Technology, Xinxiang, China; ^b^State Key Laboratory of High-Efficiency Production of Wheat-Maize Double Cropping, Xinxiang, China; ^c^Henan Key Laboratory of Hybrid Wheat, Xinxiang, China

* Corresponding authors:

Xiangdong Chen xiangdc2020@126.com


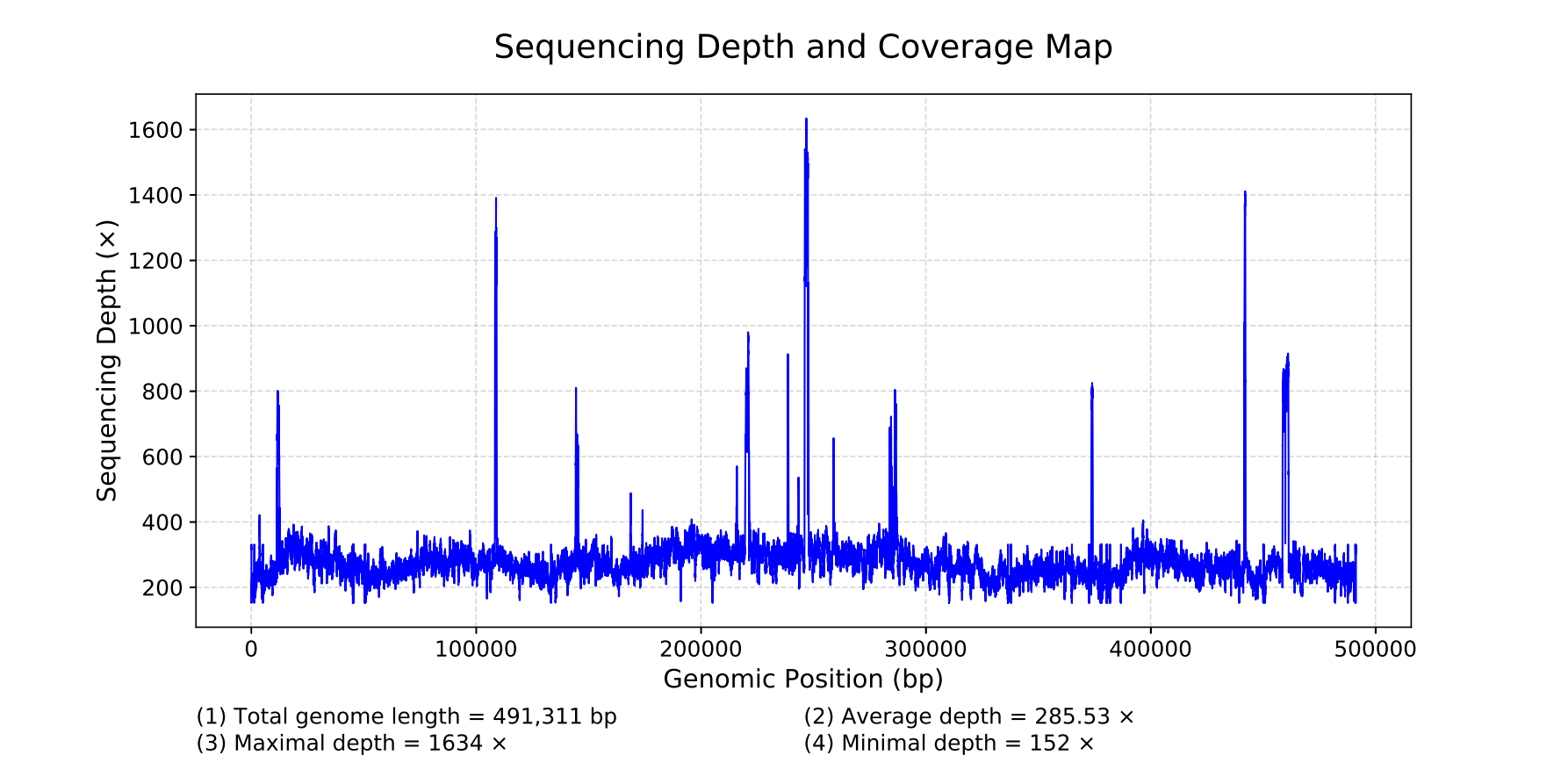


Figure S1. The overall sequencing depth and coverage map


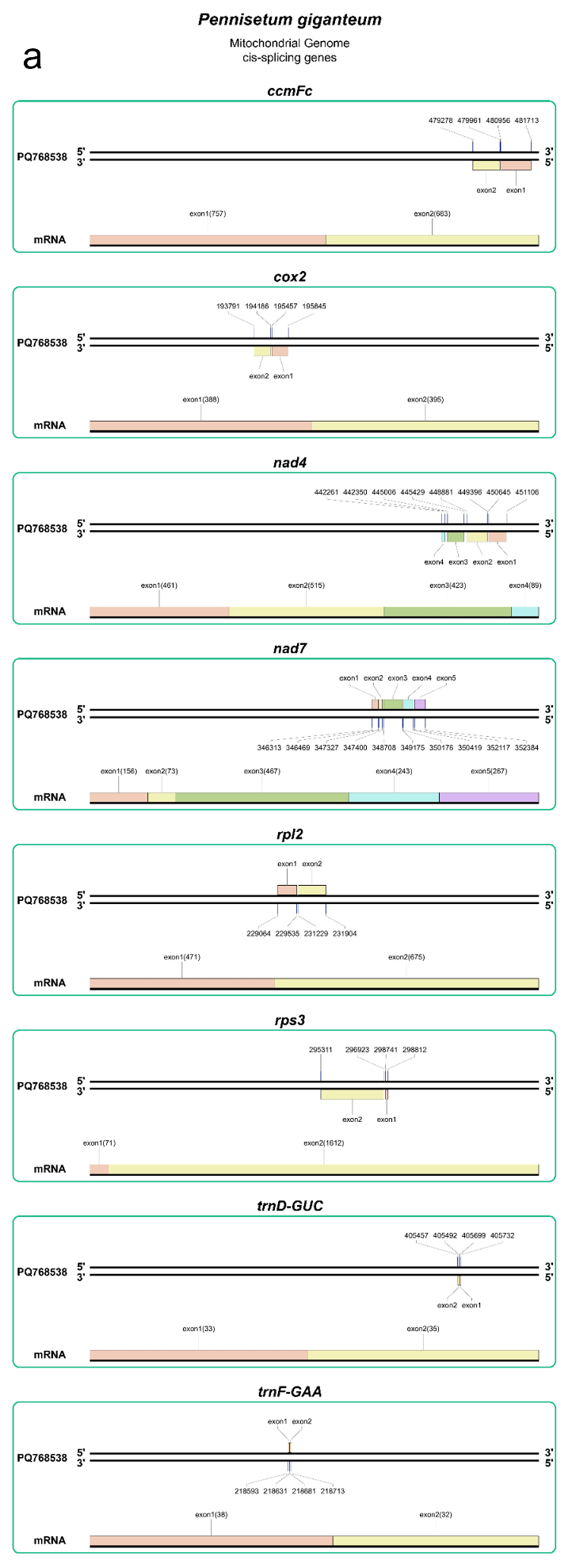


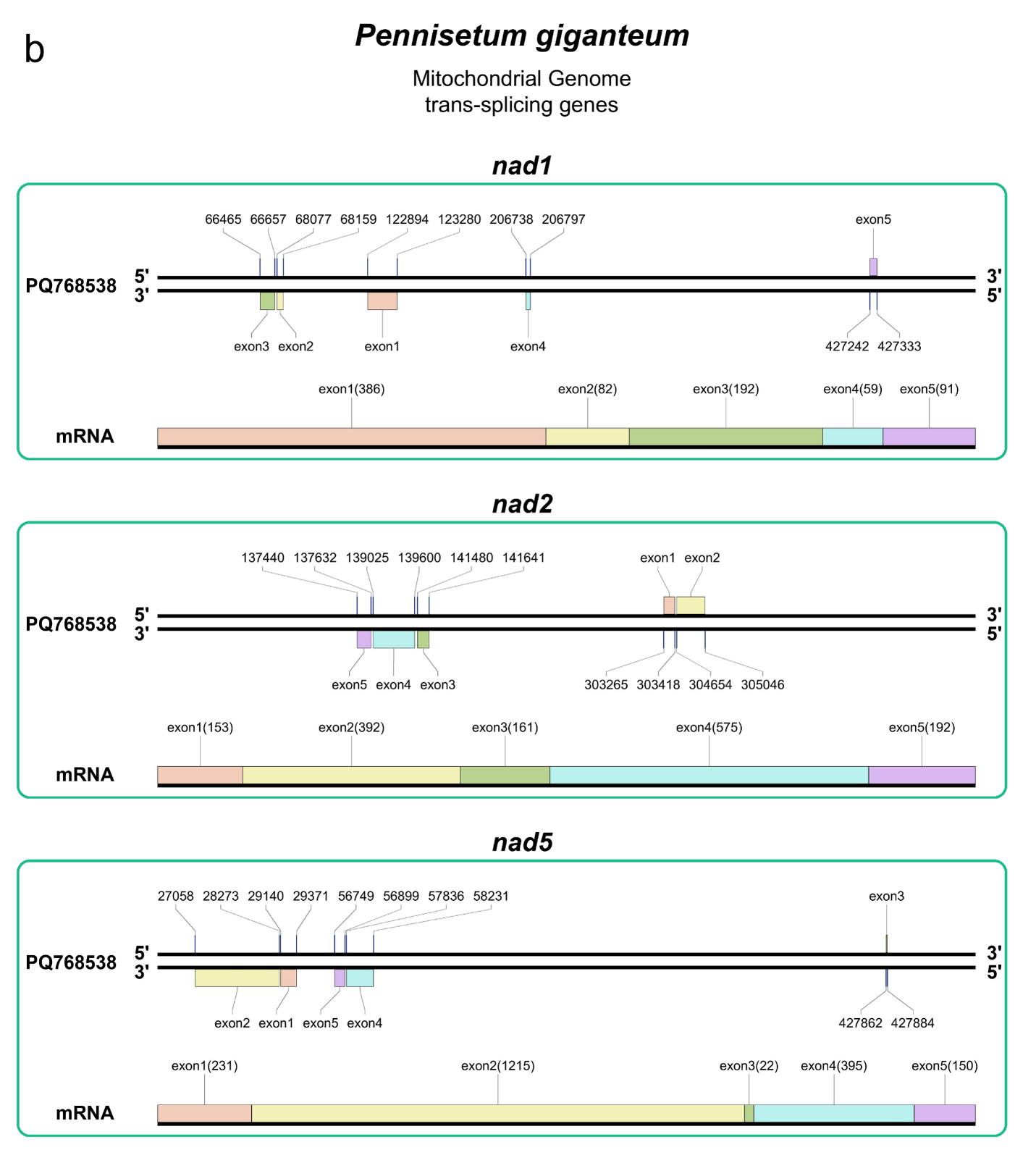


Figure S2. *Cis*-splicing gene map (a) and *trans*-splicing gene map (b) visualized in PMGmap

Table S1. Complete gene annotation of the *Pennisetum giganteum* mitochondrial genome (PQ768538)

| **Functional category** | **Groups of genes** | **Name of genes** | **No. of genes** |
| --- | --- | --- | --- |
| Electron transport and ATP synthesis | Complex_I | *nad1 nad2 nad3 nad4 nad4L nad5*  *nad6 nad7 nad9* | 9 |
|  | Complex_III | *cob* | 1 |
|  | Complex_IV | *cox1 cox2 cox3* | 3 |
|  | Complex_V | *atp1 atp4 atp6 atp8 atp9* | 5 |
|  | Cytochrome_c_biogenesis | *ccmB ccmC ccmFc ccmFn* | 4 |
| Transcription and Translation | Ribosome_large_subunit | *rpl16 rpl2* | 2 |
|  | Ribosome_small_subunit | *rps1 rps12 rps13 rps19 rps2 rps3 rps4 rps7* | 8 |
| RNA genes | Ribosomal_RNAs | *rrn5 rrnL rrnS* | 3 |
|  | Transfer_RNAs | *trnC-GCA trnD-GUC trnE-UUC trnF-GAA*  *trnH-GUG trnK-UUU trnM-CAU trnN-GUU*  *trnP-UGG trnQ-UUG trnS-GCU trnS-GGA*  *trnS-UGA trnV-GAC trnW-CCA trnY-GUA* | 16 |
| Other genes | Maturase | *matR* | 1 |
|  | Methyltransferase | *mttB* | 1 |
